# Ladies and Babies

**Published:** 1870-03

**Authors:** 


### LADIES AND BABIES.

The everlasting beauties of all ages and all climes are three: the smile of a child, the songs of the birds, and the brightness of flowers. But in a few days the flowers fade; in a single summer the birds cease to sing; but there is a loveliness in the face of childhood which survives the summer-time, and extends through the long years, numbering a score, and then care or sickness, or depravity, or all of them, make lines, and creases, and crow-feet, to deepen, and lengthen, and widen, and waste the body, and shrivel up the heart; and sorrow, and grief, and disappointment wind up the sad history.

But I have seen as smooth a brow on eighty's face, and as sweet a smile, and as merry a twinkle of the eye, as ever lighted up the countenance of babe or youth. This is rare; it ought to be oftener, it would be always if we lived aright as to body and soul; and to bring this about, we must first begin with the body, before it is born —begin with the soul before it has a mundane existence.

A good step has been taken in this direction, and as was proper and right, a woman was in the case, by name and title Mrs. Doctor Anne Dinsmore, in the establishment of a Ladies' Physiological and Sanitary Institute, to disseminate among women valuable physiological truths.

We call Boston in derision the Hub of the universe. But from that same Boston and glorious New England emanations come, modes of thought are developed, and practicalities are originated, which shoot out, and widen, and deepen, and make their everlasting impress upon the whole American mind. Boston, in more than one or two things has framed thoughts which have revolutionized the nation, which will shape the mind of the world.

In the case in hand Boston originated the idea a third of a century ago, and a society was formed in 1848, of which Mrs. W. R. Mayo was president, but it was organized amid the fierce opposition of the leading professions, and yet to-day it numbers over four hundred members. Such societies ought to be organized and to work in every part of the country, until every woman can be made to understand and to feel that it is her duty to educate herself to all the duties of motherhood, and that these duties legitimately begin on the marriage day, when the soul-character of the future child begins to be molded, and when foundations can be laid which shall give vigor of body, strength of mind, and purity of heart, to mold the destinies of the unborn for time and for eternity. It is a most momentous subject whether a child shall be born misshapen in body, feeble in mind, depraved in heart; to live a life of sickness, and crime, and misery; or to be a healthful, happy, holy being; and to be young in heart and body and mind at fourscore, depends more than we are aware of on the parents themselves, upon their thoughts, and feelings, and habits of life, during "The first year of marriage," a pregnant theme for a book. To know how to begin married life well, to know, and see, and feel what an influence it has over the individuals themselves, to last to life's latest hour, to make it a voyage of sunshine or storm to the bridal pair, and of happiness or misery to those who may be born of them, these are questions upon which the society named at the head of this article has a most natural, direct, and practical bearing, and we heartily wish it and all like it a grand success.

